# Blood Diathesis in a Patient of Rare Blood Group ‘Bombay Phenotype’

**DOI:** 10.7759/cureus.3488

**Published:** 2018-10-23

**Authors:** Sara A Awan, Ayesha Junaid, Samreen Khan, Sehreen Jahangir

**Affiliations:** 1 Hematology, Shifa International Hospital, Islamabad, PAK

**Keywords:** von willebrand disease, bombay phenotype, blood transfusion, von willebrand factor, abo blood group, von willebrand antigen

## Abstract

Bombay blood group or Oh phenotype is a rare autosomal recessive phenotype within the ABO blood grouping system. It occurs due to a mutation in the H gene that produces H antigen on red blood cells (RBCs). Individuals with two mutant H genes lack H antigen on RBCs and have anti-H antibodies in serum. At the time of blood grouping, this blood group mimics O blood group but it shows incompatibility with O group blood during cross matching. Several studies have reported an association of decreased von Willebrand factor (VWF) levels in plasma with ABO blood groups. Here we report a case of a 19-year-old male, who was labelled as Bombay phenotype and later found to have markedly reduced plasma VWF levels.

## Introduction

Bombay phenotype was first discovered by Dr. Bhende in Bombay, India and named accordingly [1]. The H antigen is located on red blood cells and is the precursor compound of A and B antigens. The A and B allele produce different transferase enzymes which add complex carbohydrates to the H antigen, transforming it into A antigen and B antigen, respectively. In individuals with blood group O there is no functional transferase enzyme to modify the H antigen, therefore it remains unchanged. The h allele is a result of the mutation of the H gene that expresses H antigen in the red blood cells. Individuals with Bombay phenotype inherit the homozygous recessive (hh) genotype instead of the homozygous dominant (HH) or heterozygous (Hh) genotypes of the ABO blood group. As the A and B antigens cannot be formed without the H antigen precursor, their red blood cells also lack these antigens. Consequently, these individuals produce anti-H, anti-A, and anti-B antibodies. Serum from these individuals contain antibodies that react with red blood cells from all O, A, B, and AB blood groups. It is of concern to note that people with Bombay phenotype can only be transfused with red blood cells that lack the H, A, and B antigens, leaving room for either autologous blood or blood from another Bombay blood group [2].

Here, we report a case where a patient with Bombay blood group presented with upper gastrointestinal bleeding and severe anemia. Once his blood group was determined, donors were reached and he received two successful blood transfusions of Bombay blood group. After stabilizing the patient, he was discharged and follow-up investigations including serum von Willebrand factor antigen, von Willebrand factor functional activity and factor VIII levels were ordered. He was consequently labelled as type 3 von Willebrand disease. This case report emphasizes the significance of both forward (cell) typing and reverse (serum) typing during ABO blood grouping in blood banks.

## Case presentation

A 19-year-old male presented in the emergency department with one episode of melena per day, for one week. It was associated with vomiting, shortness of breath and palpitations. His hemoglobin level on initial complete blood count was 5.80 g/dL, signifying severe anemia according to WHO guidelines [3]. His lab parameters on admission are presented in Table 1.

**Table 1:** Laboratory investigations on admission. RBC: Red blood cell; WBC: White blood cell; INR: International normalised ratio; APTT: Activated partial thromboplastin time; SGOT (AST): Serum glutamic oxaloacetic transaminase (Aspartate aminotransferase); SGPT (ALT): Serum glutamic pyruvic transaminase (Alanine aminotransferase).

Test	Result	Normal Reference Range
Hemoglobin	5.80 g/dL	13.5-18.0 g/dL
RBC total	2.02 m/μL	4.5-6.5 m/μL
WBC total	13900/μL	4000-10500/μL
Platelet count	474000/μL	150000-400000/μL
Prothrombin time	10.40 sec	9.5-11.7 sec
INR	1.0	0.8-1.3
APTT	48.60 sec	24.8-36.2 sec
Fibrinogen level	171.1 mg/dL	199-463 mg/dL
Factor VIII	18.5%	75-216%
SGOT (AST)	27 U/L	5-34 U/L
SGPT (ALT)	24 U/L	0-55 U/L
Alkaline phosphatase	81 U/L	40-150 U/L
Total bilirubin	0.22 mg/dL	0.2-12 mg/dL
Direct bilirubin	0.102 mg/dL	0.0-0.5 mg/dL
Gamma-glutamyl transferase	22 U/L	12-64 U/L
C-reactive protein	0.78 mg/L	Up to 5.0 mg/L
Serum sodium	141 mEq/L	136-145 mEq/L
Serum potassium	3.4 mEq/L	3.5-5.1 mEq/L
Serum chloride	105 mEq/L	98-107 mEq/L
Serum bicarbonate	24 mEq/L	22-29 mEq/L
Serum glucose(random)	133 mg/dL	<200 mg/dL
Blood urea nitrogen	4.0 mg/dL	8.9-20.6 mg/dL
Serum creatinine	0.8 mg/dL	0.72-1.25 mg/dL

Immediately packed red blood cells (RBCs) were requested from the blood bank. On forward typing his blood group was labeled as O positive and his serum showed strongly positive indirect Coomb’s test with a negative direct Coomb’s. On extended 11 cell panel antibody testing, his serum demonstrated pan-agglutination which matched with monoclonal panel cells having anti-Kell, anti-Lub, and anti-Kpb antibodies. On cross match with four O negative and four O positive packed RBCs, +4 incompatibility was seen with all. Meanwhile a detailed history of the patient revealed two distinct episodes of epistaxis in childhood and a family history of his paternal grandmother having an increased bleeding tendency. In view of his past history of fresh frozen plasma infusions, it was interpreted that the patient may have multiple alloantibodies in blood leading to gross incompatibility. Considering the urgency of the situation, one unit of the least incompatible (O negative) packed RBCs was issued after washing with normal saline thrice, to the emergency department. Transfusion was started under strict monitoring by the emergency department physicians. After slow transfusion of around 10 ml blood, the patient started shivering and his temperature spiked to 101°F with tachycardia and hypotension. The transfusion was stopped immediately and the patient was given intravenous antihistamine and hydrocortisone. Meanwhile, he was transferred to the intensive care unit (ICU) where he received intranasal desmopressin and intravenous factor VIII.

Transfusion reaction workup revealed a grade 4+ pan agglutination in his serum. During repeat blood grouping, forward typing did not demonstrate any reaction to anti-A and anti-B antisera, like a normal O blood group. However, on reverse typing, his serum showed strong agglutination with group O pooled control cells. His post saline wash incompatibility with O negative red cell concentrate showed minor difference from grade +4 agglutination (pre-wash) to grade +3 clumping (post-wash). A fresh RBCs sample from the patient showed negative direct Coomb’s test, while fresh serum sample remained positive for indirect Coomb’s test. This workup strongly raised the suspicion of Bombay phenotype and his red cells were tested with anti-H lectin, which showed no agglutination. This confirmed his blood group as Bombay phenotype. The reactions observed with Bombay phenotype compared to other blood groups, on forward and reverse typing, are illustrated in Table 2.

**Table 2:** Forward and reverse grouping with different blood groups. (+) = Agglutination, (-) = No reaction

Blood group	Anti-A antibodies	Anti-B antibodies	Anti-A,B antibodies	A1 cells	B cells	O cells
A	+	-	+	-	+	-
B	-	+	+	+	-	-
AB	+	+	+	-	-	-
O	-	-	-	+	+	-
Bombay phenotype	-	-	-	+	+	+

Immediately, voluntary donor pools were contacted in blood banks throughout the country. Overnight, a donor with Bombay negative blood group was arranged from Karachi. The packed RBCs were airlifted to Islamabad maintaining the cold chain. After crossmatching with recipient’s blood showed no reaction, the donor blood was transfused to the patient. Meanwhile, a distant relative of the patient from a nearby city, with Bombay positive blood group, consented to donate blood at our blood bank. Two days later, another unit of packed RBCs was transfused to the patient. His hemoglobin after two transfusions rose up to 7.40 g/dL. As his melena settled down on supportive therapy, an endoscopy was performed that suggested an underlying hiatal hernia. After surgical consultation, the patient was advised to reduce weight and discharged from the hospital, with a scheduled follow-up visit.

In view of the patient’s past medical history and family history, during the follow-up visit, a von Willebrand factor antigen, von Willebrand factor functional activity and factor VIII levels were ordered. His von Willebrand factor antigen level was <2.0%, von Willebrand factor functional activity was <4.0% and factor VIII level was 18.5%, consistent with type 3 von Willebrand disease. The patient and his family were counselled accordingly and referred to the hematology clinic.

## Discussion

The prevalence of Bombay phenotype is 1:10,000 in India however it is much less prevalent in the Caucasians with an incidence of 1:250,000 [4]. A study amongst an urban population in Puducherry, India demonstrated that 0.008% population had Bombay blood group and it was strongly associated with consanguineous marriage (66.66%) [5]. This prompts for pre-marital blood phenotyping for all individuals and discouragement of marriage amongst individuals carrying the h allele.

Another descriptive study spanning over a period of six years (2012–2017) comprising 36,964 blood donors at a blood bank in Bangalore, showed a prevalence of 0.005% of Bombay phenotype [6]. There has not been enough documentation of reported cases of Bombay phenotype in Pakistan. Some sporadic cases have been reported in the past, but there is a need to maintain a separate record of Bombay phenotype individuals in all leading blood banks across the country. Since this blood group is passed to offspring, one way to trace Bombay individuals is to perform thorough screening of family members and relatives of a known case. Once labelled as having Bombay blood group, these individuals should be motivated to become voluntary donors and register themselves with reference regional donation centers. Blood from Bombay blood group should only be reserved for patients with the Bombay blood group phenotype as it is extremely rare.

In the event of a surgery, where blood loss is suspected and no Bombay phenotype blood is available, acute normovolemic hemodilution may be employed. This involves removal of blood from a patient after induction of anesthesia while maintaining normovolemia with crystalloids or colloids. The blood collected is stored at room temperature in the operating room and is infused back to the patient within eight hours of collection, allowing platelets and coagulation factors to remain functional [7]. Other options may include cryopreservation of blood donated by Bombay individuals.

As individuals with Bombay phenotype are often misdiagnosed as O blood group on forward typing and reverse typing is not routinely performed in some blood banks, these individuals may be transfused with O blood group in an emergency situation. This may lead to an acute hemolytic transfusion reaction. This can be avoided by providing these patients with wrist bands or a rare blood group card, identifying their blood group. These individuals also require comprehensive counselling, emphasizing the rarity of their blood group and the need to develop a careful behaviour to avoid episodes of bleeding in the future. The importance of counselling is paramount in females where the need for blood transfusion may arise in the setting of child birth.

Von Willebrand disease (VWD) is caused by mutations that lead to an impairment in the synthesis or function of von Willebrand factor (VWF). The disease can also be acquired due to various pathophysiologic defects. It is classified as quantitative defect (type 1 and type 3) or qualitative defect (type 2). Type 2 is further subdivided into type 2A, 2B, 2M, and 2N [8]. A study observed a significant effect of ABO blood group on plasma von Willebrand antigen (VWF:Ag) levels. It demonstrated that VWF:Ag levels in patients with Bombay blood group (median VWF:Ag = 0.69 IU/dL) were significantly lower than in groups AB, A, or B. It further established that VWF:Ag levels in Bombay blood group individuals were even lower than in group O individuals (median VWF:Ag = 0.82 IU/dL), however this difference was statistically not significant [9]. Various other reports [10, 11] confirm that plasma VWF:Ag concentration is significantly influenced by the ABO blood group. In this case report too, laboratory investigations on follow-up visit demonstrated a markedly low VWF antigen level in the patient. Therefore, in dealing with a patient of Bombay blood group presenting with a history of bleeding diathesis, the suspicion of von Willebrand disease should be kept high.

## Conclusions

Bombay phenotype is one of the rarest ABO blood groups which often leads to a delayed diagnosis. It is important to perform both forward and reverse blood grouping routinely in all first time O blood group labelled patients. Once identified, these individuals should carry a form of identification of their blood group at all times. Meanwhile, their family members should be screened for Bombay phenotype and affected individuals should be counseled to become voluntary donors. Blood banks also need to create a rare group registry for prompt response in the wake of any emergency situation.
